# Educational Workshop using games improves self-monitoring of blood glucose among children*

**DOI:** 10.1590/1518-8345.2400.3039

**Published:** 2018-10-25

**Authors:** Léia Alves Kaneto, Elaine Buchhorn Cintra Damião, Maria de La Ó Ramallo Verissimo, Lisabelle Mariano Rossato, Aurea Tamami Minagawa Toriyama, Regina Szylit

**Affiliations:** 1 Faculdade das Américas, Escola de Enfermagem, São Paulo, SP, Brazil.; 2 Universidade de São Paulo, Escola de Enfermagem, São Paulo, SP, Brazil.

**Keywords:** Child, Health Education, Blood Glucose Self-Monitoring, Diabetes Mellitus Type 1, Chronic Disease, Pediatric Nursing

## Abstract

**Objective:**

to evaluate the effectiveness of an educational workshop using games to improve self-monitoring of blood glucose techniques for school children with type 1 diabetes.

**Method:**

a quasi-experimental study was conducted with school children who attended two outpatient clinics of a university hospital. Data were collected by systematic observation of the self-monitoring of blood glucose (SMBG) technique before and after the intervention. Data analysis consisted of verifying changes while performing the technique, using pre- and post-intervention compliance rates using statistical tests. The sample consisted of 33 children. Each child participated in one session; 17 educational workshops were conducted in total.

**Results:**

we found an increased frequency of SMBG, changing lancets, rotation of puncture sites, as well as calibration and periodic checking of date and time of the glucose meter. Comparisons pre- and post-intervention showed that the average number of steps in accordance with the SMBG technique increased from 5.30 to 6.58, whereas the steps “Changing the lancet of the lancing device”, “Pressing the puncture site” and “Disposing of materials used in a needlestick container” showed statistically significant differences.

**Conclusion:**

the educational workshop was effective, as it improved children’s performance of the SBMG technique.

## Introduction

Educational activities are among the most relevant interventions performed by nurses for individuals with chronic diseases. Nurses must ensure that educational strategies used with children are appropriate to their developmental stage, helping them incorporate unusual, unpleasant and even painful self-care procedures. Therefore, teaching these procedures should facilitate the child’s understanding and acceptance of his/her illness so that he/she can incorporate the treatment procedures into his/her daily life more easily. In this scenario, playful activities are suitable for the teaching-learning process. When the child is encouraged to discuss self-care procedures through games in a pleasant, safe and appropriate environment, he/she is more likely to make the necessary behavioral changes, thus increasing the child’s industriousness and self-worth^1^. The objective of this study was to evaluate the effectiveness of an educational workshop using games with school children with type 1 diabetes to improve their self-monitoring of blood glucose (SMBG) techniques.

Different studies have used playful activities as an educational strategy for self-care or for collecting data in research. However, most of them use playful strategies in the teaching-learning process to favor the transmission of knowledge about the disease or the health problem itself, rather than for performing self-care actions^2^ ^-^ ^7^. Nevertheless, knowledge alone does not change behavior. Despite the vast literature on guidelines and recommendations for the inclusion of playful strategies in childcare, little is known about the empirical results of such strategies in the teaching-learning process for the management of chronic diseases^4^. 

Among the chronic diseases affecting children, type 1 diabetes mellitus (T1DM) requires attention for its complex treatment. This disease requires specific self-care behaviors throughout the patient’s life to achieve adequate glycemic control^8^ ^-^ ^9^. A child with type 1 diabetes usually requires intensive treatment with preprandial blood glucose monitoring, resulting in numerous daily procedures to check the glycemic profile and prevent severe hypoglycemia, which is highly harmful to the nervous system^10^ ^-^ ^11^. Although considered a simple technique, blood glucose monitoring requires considerable care to ensure greater accuracy of results and to reduce the risk of infections. Glucose monitoring is the first practical self-care action performed by children with diabetes when they begin their process towards autonomy in disease management.

## Method

The research project adopted a quasi-experimental approach, in which the effectiveness of an educational workshop using games specifically made with diabetes content was tested. There were four stations with games comprising storytelling and puzzles; a bingo game; a memory game and a board game. All games contained questions related to the SMBG technique, e.g., “What must be done before pricking the finger?”, “What is the name of the device used to measure glycemia?”. The questions were repeated in different ways in each game so the children could retain the knowledge more easily. Children used each station for about 15 minutes, with another child or alone. They always interacted with the researcher. To lessen the anxiety of their parents/guardians due to fasting and its risk of hypoglycemia, participants were given a diet snack in the last five minutes of the workshop. 

The sample was selected using convenience sampling: all 36 children aged 6-11 years diagnosed with T1DM, who attended two Diabetes Outpatient Clinics of the Endocrinology Service of a university hospital in Brazil were invited. 

The inclusion criteria were:

a) Being diagnosed with type 1 diabetes for at least one year so that the child had the opportunity to experience the impact of such diagnosis and incorporate new routines into his/her life; b) Not having a diagnosis of cognitive or sensory disabilities; c) Performing self-monitoring of blood glucose at home; and d) Having the availability to participate in the two data collection phases. 

The exclusion criteria were chronic comorbidities requiring a great deal of additional care, such as cystic fibrosis and transplant.

Demographic and treatment characteristic variables were: age, gender, education level, outpatient clinic, time of diagnosis, age at diagnosis and glycated hemoglobin (HbA_1c_) value.

Participating in the educational workshop was the independent variable. Dependent variables included: a) Variables concerning the glucose monitoring profile, such as blood glucose monitoring frequency; frequency of changing the lancet; rotating fingers used for puncture; changing the device chip when beginning a new batch of test strips; periodically checking the correct date and time settings of the blood glucose meter; and b) Technical variables regarding the blood glucose monitoring steps of the procedure:

1) Thoroughly washing hands with soap and water or with 70% alcohol before pricking the finger;
2) Changing the lancet of the lancing device;
3) Pricking the lateral side of the finger;
4) Not milking the finger tip;
5) Collecting a sufficient amount of blood for the appropriate glucose reading;
6) Properly placing the drop of blood on the test strip;
7) Pressing the puncture site;
8) Checking the glycemic test result;
9) Disposing of materials used in a needlestick container.

This study was approved by the local Research Ethics Committee. All participants’ parents or guardians provided written consent, and the children gave their assent to participate in the study.

Data collection and educational workshops were held in a private room, especially prepared for the activity, on the same day of the children’s routine follow-up appointment at the outpatient clinic. Data on characterization and monitoring of blood glucose profile were collected in individual interviews with children and their guardians/parents before the educational workshop and after the workshop. Data from the SMBG technique were collected through observation while the child showed how to perform the procedure in two phases: immediately before the educational workshop and four to six weeks after the workshop. To demonstrate the SMBG procedure, 70% isopropyl alcohol pads were provided to the child and he/she was asked to demonstrate how he/she performed the SMBG at home with his/her own glucose meter, strip and lancet device. The researcher always asked the child to perform the SMBG as it was done at home and no other orientation was given in that moment. The child’s technical compliance to perform each step was assessed by registering: Compliance (C), when the procedure corresponded to the recommended standards or Non-compliance (NC). 

Data was analyzed with the Statistical Package for the Social Sciences (SPSS) version 22.0 for Windows. Compliance rates of each SMBG technique step correspond to the percentage of children who performed the step as directed. Associations between these rates and descriptive variables of the outpatient clinic, such as age and education level, were analyzed according to Generalized Estimating Equations and Linear Mixed-Effects models. To analyze the pre- and post-intervention compliance rates, McNemar’s test was used to evaluate each step, and Wilcoxon signed-rank for all steps.

## Results

We conducted 17 educational workshops using games with 36 children, with an average of two children per workshop. Three children were excluded for not attending phase two (post-intervention), for a final sample of 33 participants.

Among the 33 children, 17 (51.5%) were male; ages ranged from 6 to 11 years with age-appropriate education. Children’s illness history pre-intervention were:

a) Age at diagnosis: The minimum age was 1 year old and the maximum was 9 years old. Mean and standard deviation (SD) were 3.7 (1.9) years old and the median was 3 years old.
b) Time of diagnosis: The minimum time was 1 year and the maximum were 10 years. Mean and SD were 5.1 (2.4) years and the median was 5 years.
c) Glycated hemoglobin value: The minimum glycated hemoglobin value (HbA_1c_ - %)^1^ was 6.4% and the maximum was 16.3%. Mean and SD were 9.1% (1.8) and the median was 9.1%.

According to the Linear Mixed Effects model, the time of diagnosis showed no association with the number of correct steps pre- and post-intervention (*p*=0.252), neither with the number of correct steps independently of pre- or post-intervention (*p*=0.869). 

On the other hand, we verified an improvement in the frequency of self-monitoring of blood glucose after the intervention, as well as in changing lancets, rotation of puncture sites, calibration and periodic checking of date and time settings of the glucose meter.

Before the intervention only 18.2% of the children performed the SMBG as recommended by guidelines, which is three to four times a day. After the intervention this group increased to 27.3%. In addition, the percentage of children that performed the SMBG once to twice a day decreased from 6.1% to 3.0%. The group that performed the SBMG five or more times a day decreased from 75.7% to 69.7%.

**Figure 1:**
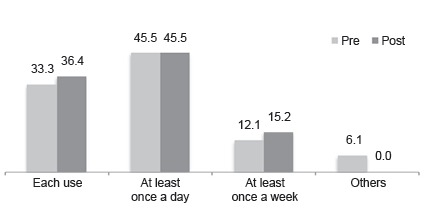
Percentage of children according to frequency of changing the lancet, pre- and post-intervention (n=33).

Figure 1 presents the distribution of children according to the frequency of changing lancets, showing that the main behavioral change occurred among those who only sporadically or never changed lancets. 

Table 1 shows an overall increase in the performance of all procedures that contribute to the accuracy of blood glucose test results. 

**Table 1:** Proportion of school children with type 1 diabetes mellitus who perform actions/ procedures that contribute to the accuracy of blood glucose test results (n=33). São Paulo, SP, Brazil, 2015

Practices	Pre-Intervention	Post-Intervention
Rotating fingers used for puncture	84.8 %	100 %
Changing the device chip when starting a new batch of reagents	93.9 %	100 %
Periodically checking the correct date and time settings of the glucose meter	78.8 %	93.9 %

The educational workshop was efficient as it changed children’s behavior: one more step was conducted in accordance with the recommendations in the post-intervention period, regardless of the child’s education level (Table 2). 

**Table 2:** Number of steps performed in accordance with the blood glucose monitoring technique and standard deviation pre- and post-intervention, considering the total number of children and their education level (n=33). São Paulo, SP, Brazil, 2015

		Mean (SD*)		*p*-value
		Pre-intervention	Post-intervention	
	Total children	5.3 (1.6)	6.6 (1.2)	0.001^†^
Total steps performed in accordance with the recommendations	Education			
	First grade	4.5 (1.9)	6.0 (1.2)	0.526^‡^
	Second grade	4.7 (2.5)	7.0 (1.4)	
	Third grade	4.7 (1.6)	6.2 (2.2)	
	Fourth grade	6.4 (0.8)	7.0 (1.2)	
	Fifth grade	5.9 (1.1)	6.6 (0.8)	
	Sixth grade	4.6 (1.5)	7.0 (1.0)	

*SD: Standard Deviation; †Wilcoxon Signed-Rank Test; ‡Linear Mixed Effects Model.

**Figure 2:**
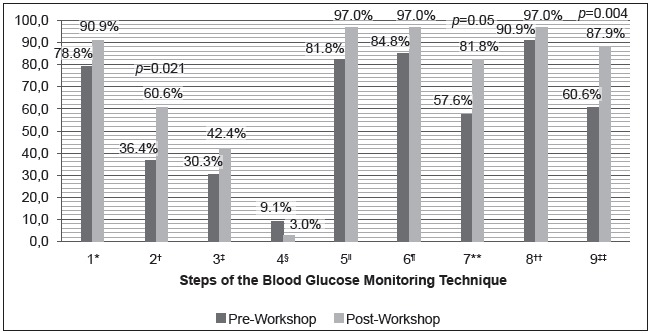
Comparison of compliance rates pre- and post-intervention according to the steps of the Blood Glucose Monitoring Technique

Figure 2 shows an improvement in all compliance rates of the SMBG pre- and post-intervention, except for the fourth step (Not milking the finger tip). The overall compliance rate of the SMBG technique in the pre- and post-intervention periods was 0 (zero), since children did not achieve 100% compliance with all steps.

Caption: 1* Thoroughly washing hands with soap and water or 70% alcohol before pricking the finger; 2† Changing the lancet of the lancing device; 3‡ Pricking the lateral side of the finger; 4§ Not milking the finger tip; 5|| Collecting a sufficient amount of blood for the blood glucose reading; 6¶ Properly placing the drop of blood on the test strip; 7** Pressing the puncture site; 8†† Checking the glycemic test result; 9‡‡ Disposing of materials used in a needlestick container.

We also observed an improvement in compliance rates when children were analyzed according to age and education level (data not shown). Only the step “Not milking the finger tip” presented some improvement among second graders; however, worse results for this step were found in all other groups, with no significant differences. 

In short, the educational workshop using games was efficient in helping children to perform an additional step of the SMBG technique and increase the frequency they changed the lancet and checked the adequacy of the blood glucose meter settings. Furthermore, we found 100% compliance regarding the rotation of the puncture site and calibration of the glucose meter. 

## Discussion

This study tested the effect of a strategy, the educational workshop using games, to improve self-care practices of school children with T1DM, this disease was considered as an example of a situation in which children need to incorporate unusual, unpleasant and even painful procedures into their lives. The educational workshop using games was considered by the researchers as a potentially favorable strategy to help children understand and accept these procedures. 

We must highlight that all children enjoyed doing the activities and showed interest in participating. They remained throughout the workshop, interacting with the researcher and the other kids. 

The mean time of diagnosis shows that most children in the study had been living with T1DM for a long time. In this sense, they had already been performing SMBG; therefore, the objective of the study was to evaluate their performance and observe possible improvements after the educational workshop using games. 

The mean glycated hemoglobin value was high, indicating that control values were above the recommended, which is <7.5%^12^. Several factors may lead to this result, such as non-compliance with the SMBG technique, which was also found in this study (Table 2 and Figure 2)^12^ ^-^ ^13^. Verifying compliance rates for self-monitoring, we observed important situations that may have caused misleading blood glucose test results, which in turn led to the incorrect choice of measures for blood glucose control that may have contributed to high glycated hemoglobin values^13^.

All children were under intensive treatment regimen. Therefore, they were supposed to perform blood glucose monitoring three to four times a day, before the main meals and before sleeping, following the recommended steps. Prior to the workshop most children conducted the procedure at different frequencies, some more than five times a day, but none was in accordance with the technique. In fact, higher frequencies of SMBG, above the recommended standards, may have been the result of the child’s family anxiety^14^ ^-^ ^15^.

Our results showed a positive effect of the educational workshop using games on blood glucose monitoring: compliance, which was low, with 5.3 steps performed on average, increased after a single workshop. It is well known that diabetes education should be a continuous and long-term process^9^. This may explain that inspite of the improvement reported, compliance remained below the desired level with 6.8 steps performed, thus supporting the importance of regular educational interventions to ensure better self-care practices^16^ ^-^ ^18^.

We must highlight that three steps of the technique presented significant improvement after a single intervention session: “Changing the lancet”, “Pressing the puncture site”, and “Disposing of materials in a needlestick container”.

Regarding the “Changing the lancet” step, reusing the lancet is controversial. Lancets are known to be a disposable single-use material to avoid the risk of infections^19^, it is also known that repeated use makes it blunt, which may lead to the child refusing to perform blood glucose monitoring as pricking the finger becomes increasingly painful^20^ ^-^ ^21^. However, in the absence of the product, and considering the low incidence of infections described in the puncture site, reusing the lancet is acceptable. There are no established recommendations regarding reuse, in fact, in some areas of developing countries reusing the lancet is necessary. Therefore, studies are required to regulate its reuse and to assess the risks and benefits of this practice. Given this scenario, different orientations are being followed in the absence of standardized recommendations^21^. 

The step “Pressing the puncture site” ensures homeostasis of the punctured site, thus decreasing the risk of infection, preventing leakage of blood into the surrounding tissue, and decreasing pain and sensitivity on the fingertip^20^ ^,^ ^22^.

The step “Disposal of biological waste” presented significant differences. Patients with type 1 diabetes need guidance on how to dispose of materials used in needle stick containers; this procedure is directly linked to caring for the environment and society^23^ ^-^ ^24^.

Improvements were found in the step “Thoroughly washing hands thoroughly with soap and water or 70% alcohol before pricking the finger”, but with no statistically significant difference. We must highlight that this procedure has a high impact on the results of blood glucose tests.

The step “Not milking the finger” had the lowest compliance rate, and after the interventions results were even worse, despite all the guidance to not do so. The habit of milking the finger can be found in the literature^25^, but there are no studies investigating the causes for such practice after lancing the finger during the SMBG.

Some information that may be relevant to understand the effectiveness of the educational workshop using games was not systematically collected since that was not the focus of the study. Children’s behavior during the activities showed that they felt very confident when reporting procedures that were not in accordance with the standards and the reasons for doing so. For example, regarding the inadequate frequency to change lancets, most children replied that its reuse was not due to an insufficient quantity of the product, but to obliviousness. Some children also mentioned that they were aware of the need to change it, but that they did not understand why they had to do it. Several parents/guardians also reported that many children started performing the SMBG spontaneously and more frequently after the workshop. These facts confirm the assertion that knowledge alone does not change behavior, given that the children already knew how to properly conduct the procedure. Knowledge does not guarantee that they will actually do it. Thus, we believe that the workshop achieved its goal as it also mobilized emotional aspects, not only cognitive dimensions. 

The strength of this study was in highlighting the positive impact of a single educational session on daily behaviors in children’s routine. The small number of participants can be considered a limitation, so generalizations must be made with precaution. Further studies are required to identify the number of workshop sessions that will help children to incorporate the SMBG technique 100% correctly into their routine. In addition, it is recommended that future studies examine other indicators of effectiveness, such as the involvement of children in the educational process.

## Conclusion

Diabetes education is the most important part of the care for children with type 1 diabetes. Pediatric nurses are the professionals who take care of the children with diabetes in all scenarios, such as in Pediatric Intensive Care Unit wards and outpatient clinics. In this sense, these professionals have a great opportunity to provide diabetes education for children and their parents.

This study confirmed that the use of games as an intervention may be helpful in teaching and improving compliance for the SMBG technique. This study also shows the importance of nurses incorporating playful resources into their child care practices, and that the strategies used stimulated the child’s autonomy and proactiveness in his/her self-care. Additionally, the game intervention can be applied to other chronic diseases, especially those that require daily self-care practices, such as asthma with its use of inhalers or the peak flow meter.

## Footnotes

1 Recommended values for children: HbA_1c_<7.5%. American Diabetes Association. Children and Adolescents. Diabetes Care. 2017;40(Suppl.1): S105-S13.
